# A Cognitive Model for Aggregating People's Rankings

**DOI:** 10.1371/journal.pone.0096431

**Published:** 2014-05-09

**Authors:** Michael D. Lee, Mark Steyvers, Brent Miller

**Affiliations:** Department of Cognitive Sciences, University of California Irvine, Irvine, California, United States of America; University of California, San Francisco, United States of America

## Abstract

We develop a cognitive modeling approach, motivated by classic theories of knowledge representation and judgment from psychology, for combining people's rankings of items. The model makes simple assumptions about how individual differences in knowledge lead to observed ranking data in behavioral tasks. We implement the cognitive model as a Bayesian graphical model, and use computational sampling to infer an aggregate ranking and measures of the individual expertise. Applications of the model to 23 data sets, dealing with general knowledge and prediction tasks, show that the model performs well in producing an aggregate ranking that is often close to the ground truth and, as in the “wisdom of the crowd” effect, usually performs better than most of individuals. We also present some evidence that the model outperforms the traditional statistical Borda count method, and that the model is able to infer people's relative expertise surprisingly well without knowing the ground truth. We discuss the advantages of the cognitive modeling approach to combining ranking data, and in wisdom of the crowd research generally, as well as highlighting a number of potential directions for future model development.

## Introduction

People have all sorts of different knowledge, and are able to express their knowledge in many ways. One ubiquitous form and expression of knowledge involved *ranking* or *ordering* items to produce a structured *list*, giving the relative positions of a set of items with respect to a criterion of interest. Members of a selection panel might each rank the job candidates they have just interviewed from best to worst, children offered the choice of three fast food restaurants can quickly communicate a preference order, and popular culture generates new “top 10” lists every day.

In this paper, we consider the well-known *wisdom of the crowd* effect [1] applied to rankings. The wisdom of the crowd effect involves combining or aggregating the knowledge expressed by different people, and considering how the aggregate and individual expressions perform relative to some goal or criterion. It is often found that the aggregate of a set of estimates—of the number of jelly beans in a jar, or of the temperature in a room, or of the final margin of a sporting contest—is closer to the truth than most or all of the individual estimates from which it is calculated. Thus, our focus is on combining the rankings provided by different people in those situations where there is a (current or future) ground truth against which an aggregate ranking can be assessed.

One motivation for considering ranking data in the wisdom of the crowd context is that lists are a particularly powerful expression of knowledge, because of their combinatorial nature. If 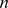 items are ranked, one answer has been identified out of 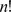 possibilities, which, for even moderately large 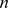, is very informative. Ordering 10 items constitutes making a single selection from 3,628,800 alternatives. A second motivation is that producing relative rankings is often easier than providing absolute numerical estimates, and the potential loss in detail may be compensated by reliability. People can more easily and reliably order the cities New York, Chicago, San Diego and Nashville in terms of their relative populations than they can estimate the actual populations. If the knowledge that is sought is satisfied by an accurate rank order, it makes sense to deal directly with those orders, rather than derive them from sorting population estimates that are more difficult to obtain.

The wisdom of the crowd effect for ranking data has been studied since at least psychology experiments in the 1920s [2]. Much of this previous research relies on statistical methods for aggregating rankings, such as the Borda count [3]. The essence of these statistical approaches is that each item is given points for the position it is placed in individual rankings, and a combined points tally is sorted to produce an aggregate ranking. Many probabilistic models for analyzing and aggregating rank data have also been developed in the statistics, social sciences, and machine learning communities [3], [4]. In the social sciences, rank aggregation methods are often applied to the problem of combining people's preferences. In machine learning, methods for aggregating rankings have found application in problems as diverse as combining the lists returned by multiple search engines [5]–[7], and the lists produced by different algorithms for object tracking [8], [9].

In this paper, we develop, demonstrate and evaluate an aggregation approach motivated by modeling the psychological process by which people produce ranking data. Theoretically, our approach differs from most previous methods because it assumes the existence of a ground truth and the presence of meaningful individual differences in the knowledge of different individuals. These two assumptions form the foundation of our model, unlike preference models that assume there is no ground truth, and statistical methods for aggregation that do not explicitly incorporate individual differences. Methodologically, our approach differs from many previous ones by relying on a *generative* model that formalizes the joint distribution between data and model parameters, and so is able to operate in a completely unsupervised way [5].

The outline of the paper is as follows. In the next section, we describe and implement a model—based on classic representational ideas in psychology dating back to Thurstone in the 1920s [10]—for generating rank order data from latent knowledge with individual differences. We then describe a corpus of data sets in which people rank order items, dealing with various domains, collected in a variety of ways, and involving both general knowledge and prediction tasks. We present the results of applying the model to all of these data sets, and compare its performance to individual performance and the Borda count. Finally, we discuss the strengths and directions for extension of our approach, and its implications for integrating wisdom of the crowd methods for aggregating knowledge with psychological models.

## Thurstonian Model

The basic assumptions of a Thurstonian model in psychology is that the attributes of stimuli can be modeled in terms of a psychological continuum, represented by coordinates on a latent dimension, and that there is variability associated with these representations [10], [11]. In its application to the wisdom of the crowd effect, the natural assumption is that the underlying dimension corresponds to the criterion of interest, the coordinate locations correspond to the ground truth, and the variability in the representations corresponds to the uncertainty people have about the ground truth.

**Figure 1:**
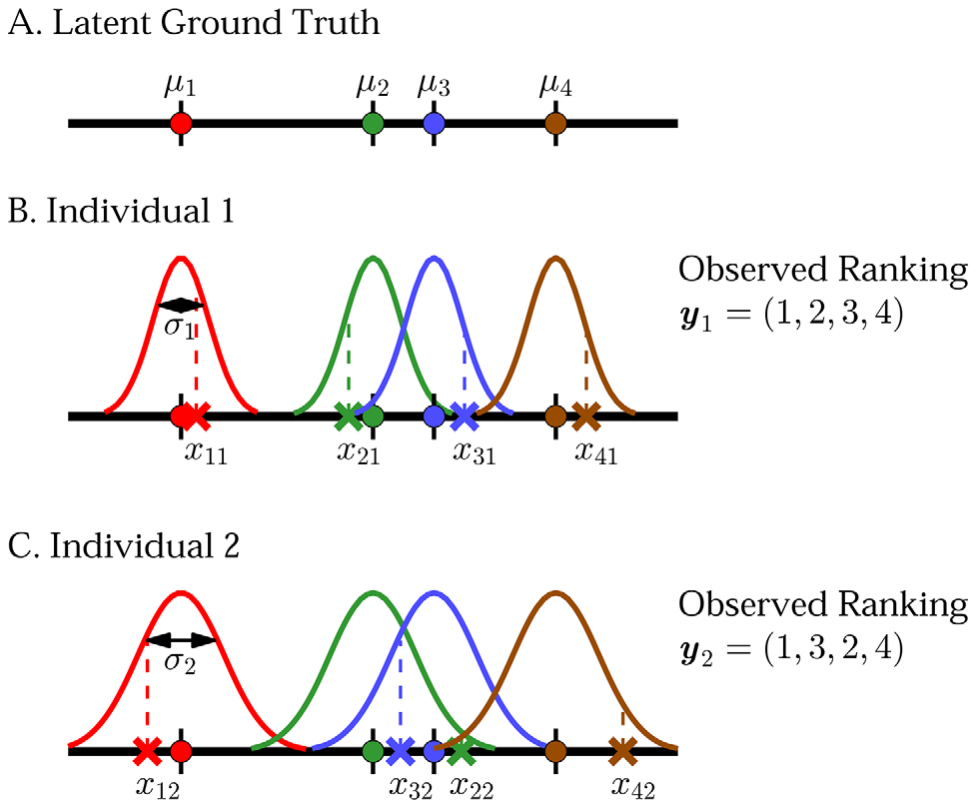
Illustration of the basic Thurstonian model. Panel A shows the latent ground truth locations 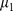, 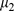, 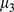, and 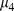 of four items. Panels B and C show, for two individuals, how the latent representations generate the mental samples that produce observed ranking data. The mental samples 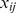 for the 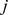
th individual on the 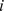
th item are draws from the Gaussian distribution with mean given my the ground truth 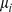 for the item, and standard deviation 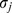 for the individual. The ordering of these specific mental samples produces the reported ranking 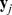 for the 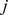
th individual.

Figure 1 provides a conceptual overview of our Thurstonian model [12], [13], using a simple example involving four items and two individuals. Panel A shows the latent representation for the three items, 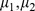, 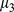, 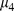 on a scale. Panels B and C show how these items are represented by two individuals, and how these representations generate their ranking data.

The model assumes that the means of the item distributions 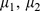, 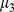, 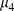 are the same for everybody, corresponding to the idea that there is a single underlying truth to which everybody has (imperfect or incomplete) access. In an extension of the standard Thurstonian approach, however, we allow the widths of the distributions to vary. This generalization allows the model to accommodate the assumption that there are individual differences in the knowledge about items. In Figure 1, Individual 1 is shown as having more precise knowledge than Individual 2, and so 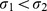.

To determine their ranking of the items, our model assumes the 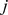
th individual takes a mental sample for all of the items. For the 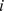
th item, this sample, 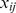, is drawn from a Gaussian distribution with mean 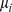 and standard deviation 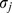. The order of the mental samples then determines the ranking 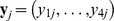 produced by the individual, where 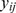 is the rank position in which the 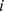
th item was placed. In the example in Figure 1, the specific samples drawn by Individual 1 lead to the ranking 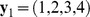 whereas Individual 2, in panel C draws a sample for the third item that is smaller than the sample for the second item, leading to the ranking 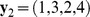. More generally, the difference in the 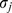 values means that 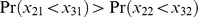, and so Individual 1 is much more likely than Individual 2 to rank items two and three in the correct order. In this way, the overlap in the item distributions can lead to differences the orderings produced by individuals, and errors with respect to true rankings.

The parameters of the Thurstonian model permit two key analyses. One analysis uses the 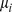 parameters, which represent the location of each item along the assumed latent dimension. The inferred ordering of the 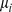 parameters corresponds to an aggregate or group ordering that has combined the ranking data provided by the individuals. A second analysis involves the 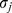 parameters, which, since they measure the precision of knowledge about the items, correspond psychologically to the expertise of each individual.

## Methods

### Ethics Statement

The data used in this study were collected in four experiments approved by the University of California Irvine Institutional Review Board in human subjects protocol HS#2009–6757. Informed consent was obtained through the presentation of a study sheet, followed by verbal acceptance (for experiments done in person) or through clicking “accept” rather than “decline” (for online experiments). These forms of consent are appropriate, and approved by the by the University of California Irvine Institutional Review Board, because of the determined Exempt status of the research procedures.

### Participants

The participants for all of the experiments were undergraduates recruited from the human subjects pool at the University of California Irvine, and were compensated by course credit. In the third and fourth experiments, participants were allowed to choose whether or not to complete some of the questions. This means that there are different numbers of participant for the tasks within these experiments. In particular, a few of the tasks that were unpopular have many fewer participants. The exact number of participants who completed each task is reported in the Tasks section below. Some questions used in the second experiment were also used in the third experiment, and so the two sets of participants have been combined for analysis.

It is possible to express the behavioral data either in an *ordering* representation, in which the order items were placed is listed in sequence, or a *ranking* representation, in which the position of each item is recorded in sequence. These representations convey the same information, and it is easy to translate between them. All of the raw data for every experiment are available, in the ordering representation, at http://webfiles.uci.edu/mdlee/LeeSteyversMiller2014Data.zip.

### Procedures

All of the experiments involve ranking tasks requiring both general knowledge of existing ground truths and predictions of future outcomes. In all cases, every participants provided a complete ranking of every item. The first experiment collected data using physical cards for each item that were physically placed in order by the participant on a large table and recorded manually by the experimenters. The second, third, and fourth experiments used a computerized experimental interface, in which the items were originally placed in a random order, but could be dragged and dropped until the participant was ready to submit their ranking and move to the next task. No time limit was imposed on the completing any of the tasks.

### Tasks

The first experiment involved 26 participants completing a single task that required ranking the 44 US presidents in chronological order.

The second experiment involved 78 participants completing a set of 17 ranking tasks. All of the tasks used 10 items and involved general knowledge, but varied with respect to the nature of the criterion for ranking. The tasks variously involved criteria relating to time (US presidencies, US holidays, movie releases, classic Oscar-winning movie releases, recent Oscar-winning movie releases, book releases, and Superbowl appearances), population (US cities, European cities, world cities, and countries), geography (US state locations, country landmasses, and river lengths), physical properties (hardness of materials), political facts (the ten amendments), and religious conventions (the ten commandments).

The third experiment involved a maximum of 70 participants completing a set of 8 tasks, 5 of which (US presidencies, US holidays, US cities, world cities, and country landmasses) were the same 10-item tasks as for the second experiment, and one of which (nine of the ten amendments, combining “trial by jury” and “civil trial by jury”) was almost the same. The other two tasks in the third experiment were prediction tasks, for the 32 teams in the US National Football League (NFL), and the 21 competitors in the US reality television show “Survivor: Nicaragua”.

The fourth experiment involved a maximum of 148 participants completing two prediction tasks, relating to the Western and Eastern conferences of the US National Basketball Association (NBA).

## Results

We applied the cognitive model to all of the data sets by implementing it as a graphical model in JAGS [14], based on a recently developed approach for related Thurstonian models [15]. We used flat and relatively uninformative priors on the 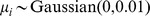 latent location and 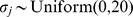 expertise parameters. Note that, as is the convention in JAGS, we parametrize the Gaussian distribution in terms of a mean and precision (not variance). Our modeling results are based on collecting 4 independent chains with 1000 samples each, after 10000 samples of discarded burn-in samples and thinning each chain by recording only every 10th sample. The chains were checked visually for lack of auto-correlation, and their convergence was assessed using the standard 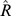 measure of within-to-between-chain variability [16]. Further formal details of both the Borda count method, and the Thurstonian modeling method, are provided in Appendix S1.

### The 44 US Presidents Task

**Figure 2:**
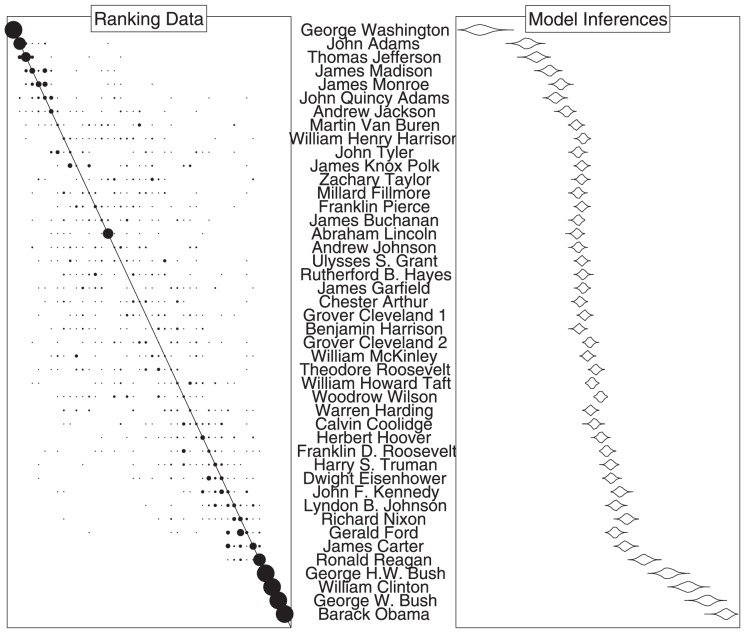
Data and model inference summaries for rankings of the 44 US presidents. The presidents are presented in true chronological order from top to bottom. The left “ranking data” panel shows the proportion of times each president was ranked in each position across 26 participants. The right “model inferences” panel shows, using a simple violin plot approach [29], the marginal posterior distribution of the parameters of the Thurstonian model. These posterior density distributions represent the location of each president on an assumed latent dimension corresponding to their chronological order.

Figure 2 presents some aspects of the empirical and modeling results for the US presidents data from the first experiment. The “ranking data” panel shows, by the areas of the circles, the proportion of times each president was ranked in each position. It is clear people have more accurate knowledge of the earliest presidents (Washington, Adams, …) and the most recent presidents (Clinton, Bush, Obama, ..). It is also clear that many people know Lincoln was the 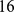
th president, even though knowledge of nearby presidents is much worse.

The “model inferences” panel shows the marginal posterior distribution, and its expectation, for each of the 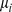 model parameters, summarizing the inferred location of each president on the assumed latent dimension. The ordering of these parameters corresponds to the group ordering inferred by the model, so the probability 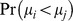 corresponds to the probability the 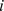
th president is ranked before the 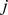
th president. It is clear, for example, George Washington is near-certain to be ranked first in the group's answer, but that Ford may mistakenly be placed before Nixon. In general, the increase in the inferred 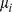 parameters with respect to true chronological order suggests that the group's answers will often be reasonably good ones.

**Figure 3:**
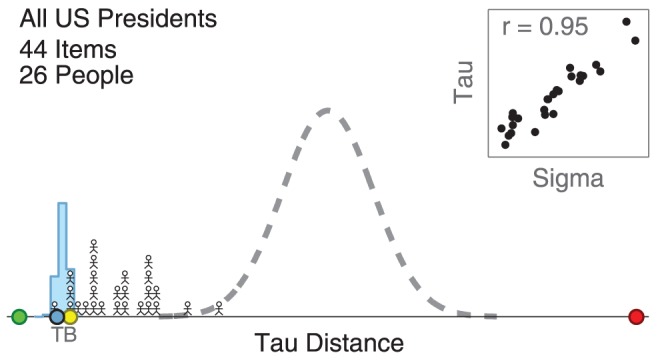
Performance of participants and aggregation methods for the 44 US presidents task. The distribution of tau distances between participants' rankings and the true order is shown by stick figure people, relative to best-possible (green circle), worst-possible (red circle), and chance (dotted line) performance. The distribution of tau distance for ranking inferred by the Thurstonian model is shown by the blue histogram, and the performance of a single ranking produced by the Thurstonian is shown by a blue circle labeled “T”. The performance of the ranking produced by the Borda count method is shown by the yellow circle labeled “B”. The inserted scatter-plots shows relationship between the inferred parameter measuring the expertise individual participants and their tau distance measure of actual performance.

Figure 3 presents an analysis that quantifies the relationship between the rankings inferred by the model and the rankings provided by the participants. It relies on Kendall's tau, which counts the number of adjacent pairwise swaps between two orders, to measure performance [17]. The green marker on the left indicates a tau distance of zero, corresponding to an answer matching the true order. The red marker on the right indicates the largest possible tau distance, corresponding to an answer that reverses the true order. The dotted line shows the chance distribution of tau distance, corresponding to the performance of orders generated at random.

The distribution of tau distance for the 26 participants is shown by the histogram of stick figures. The distribution of tau distance for the Thurstonian model is shown by the blue histogram. A distribution is needed because the model does not produce one ranking. Rather, every sample from the joint posterior distribution of 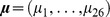 corresponds to a ranking, and the blue histogram in Figure 3 shows the tau distance for each possible ranking, weighted by its posterior mass. The blue circle labeled “T” indicates the tau distance for a single model ranking, corresponding to the ordering of the marginal posteriors for each 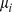 parameter. The yellow circle labeled “B” shows the tau distance for the ranking generated by the Borda count method.

It is clear from Figure 3 that participants performed above chance, but that there were large individual differences in the accuracy of their rankings. Both the Thurstonian model and the Borda count rankings performed as well as the very best participants, with the single ranking produced by the Thurstonian model being slightly closer to the truth than the Borda count ranking.

Figure 3 also shows, in the inserted scatter-plot, the relationship between the 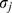 expertise parameter and actual performance over all of the participants. Each participant is a point, plotted in terms of the posterior mean of their 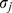 and their 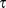 distance from the true ordering. There is a strong positive correlation of 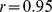 between these measures.

### General Knowledge Tasks

**Figure 4:**
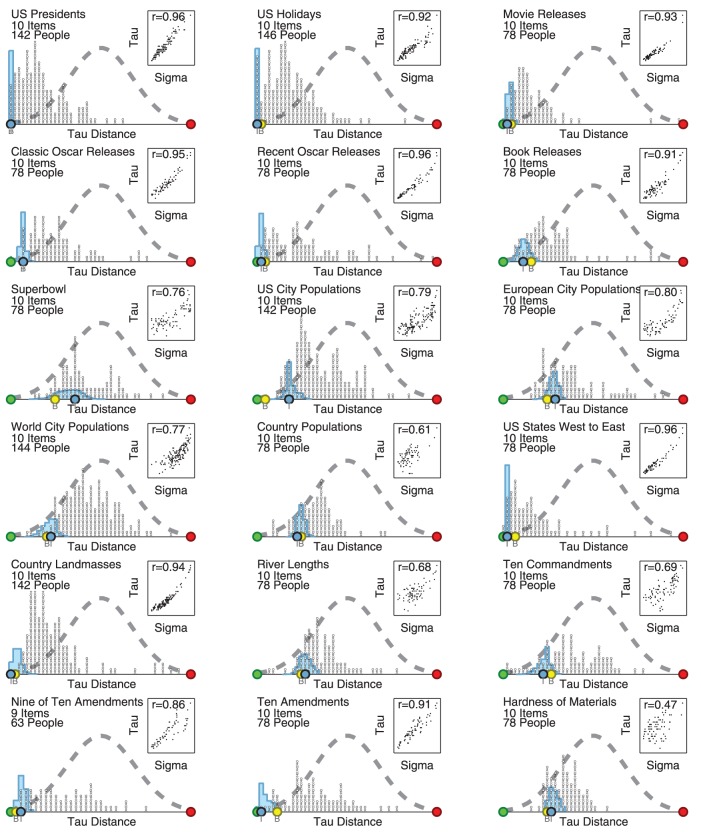
Performance of participants and aggregation methods for 18 general knowledge ranking tasks. Each panel corresponds to a different task, and shows the distribution of the tau distance measure of performance for all participants (stick figure people), the Thurstonian model (blue histograms) and summaries of the Thurstonian model (blue circle) and Borda count (yellow circle) aggregation. The inserted scatter-plots show the relationship between the inferred expertise parameter and actual performance across all participants.

Figure 4 presents the results for the remaining 18 general knowledge tasks, using the same display approach introduced in Figure 3. For all of the tasks, it is clear that there are significant individual differences in the performance of participants. For some tasks (e.g., US holidays, movie releases, country landmasses) the distribution of participant's performance is well above chance performance. For other tasks (e.g., world city populations, European city populations, hardness of materials) it is far less impressive.

Figure 4 shows that, for almost all the tasks, the Thurstonian model performs as well or better than most participants. For some tasks (e.g., US presidents, US holidays, recent oscar releases, US states east to west, country landmasses, ten amendments) the model's inferring ranking is perfect or nearly perfect. In other tasks (e.g., book releases, country populations, river lengths) the model's ranking fares well relatively to most participants, but does not match the ground truth. In just a few tasks (e.g., Superbowl appearances, European city populations, ten commandments) the ranking inferred by the model is significantly worse than some sizable subset of participants. The performances of the rankings produced by the Thurstonian model and by the Borda count are usually similar. There are only a few tasks for which either the Borda count or Thurstonian model (e.g., US city populations) is clearly superior.

Finally, Figure 4 shows that the expertise for each participant inferred by the Thurstonian model almost always has a strong correlation with the tau distance measure of performance. Many of the correlations are above 0.90, and the lowest correlation is 0.47 for the hardness of materials task. The scatter-plots show that these correlations are not being generated by outliers, but summarize what is generally a close linear relatonship between model-inferred expertise and actual observed performance.

### Prediction Tasks

**Figure 5:**
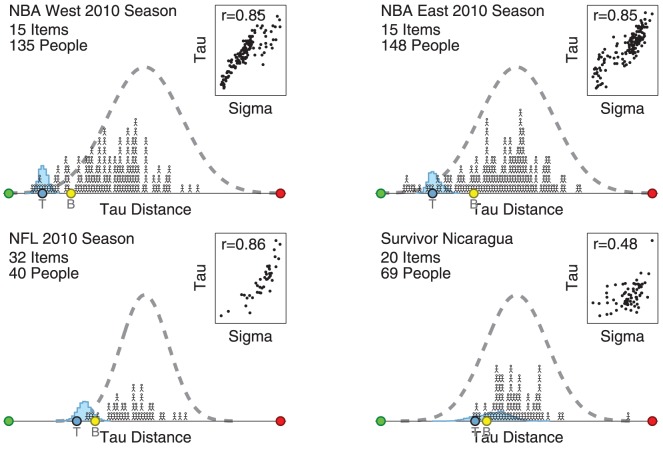
Performance of participants and aggregation methods for 4 prediction tasks. Each panel corresponds to a different task, and shows the distribution of the tau distance measure of performance for all participants (stick figure people), the Thurstonian model (blue histograms) and summaries of the Thurstonian model (blue circles) and Borda count (yellow circles) aggregated rankings. The inserted scatter-plots show the relationship between the inferred expertise parameter and actual tau distance performance across all participants.

Figure 5 presents the results for the 4 prediction tasks, using the same display approach. Significant individual differences in the accuracies of the rankings predicted by participants are again evident. For these tasks, the distribution of participant performance generally does not seem much better than expected by the chance distribution. The aggregate rankings inferred by the Thurstonian model are always among the best-performed participants, and for the NFL task outperforms all participants. The aggregate rankings determined by the Borda count method also perform relatively well, but are not as impressive, and are never as good or better than the Thurstonian rankings. For three of the four tasks (all but Survivor Nicaragua, where the correlation is 0.48) there is a strong correlation around 0.85 between the inferred expertise of participants and their tau distance measure of actual performance.

### Overall Performance

**Figure 6:**
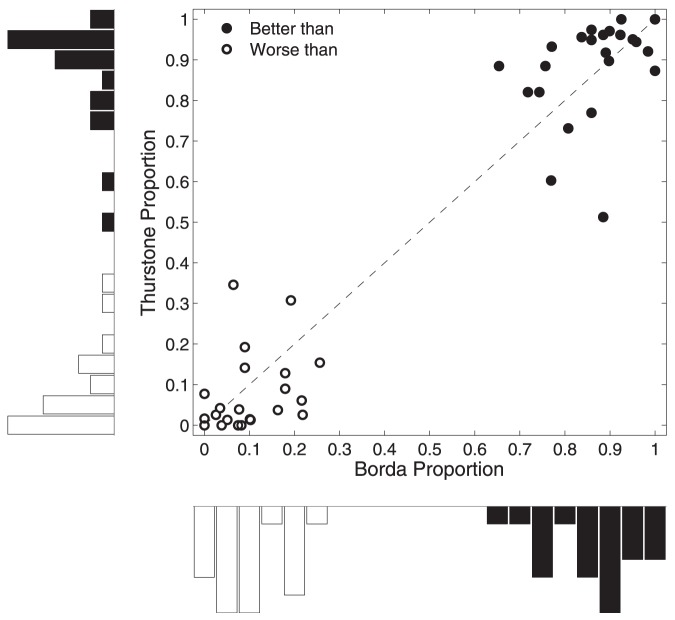
Summary of the performance of the Thurstonian model and Borda method for all task. Each point in the main panel corresponds to a task, showing the proportion of participants each method was better than (filled circles) and worse than (open circles). The histograms show the marginal distributions of these measures.

Figure 6 presents a summary of the Thurstonian model and the Borda method over all of the tasks. For these analyses, the single ranking produced by the Thurstonian model was compared to the single ordering produced by the Borda count method. The main panel shows the proportion of participants each method was strictly better than, and strictly worse than, according to the Kendall tau metric. Using just one of these measures is not complete, because it is possible for a method to have the same level of performance as participants on a task. The marginal distributions of these proportions for both methods are also shown.

One clear conclusion from Figure 6 is that both the Thurstonian model and Borda method perform well. They are typically better than 80% or 90% of participants, and usually worse than only 10% or 20%. A second, more subtle, conclusion is that the Thurstonian model usually performs better than the Borda method. Most of the tasks lie above the diagonal for the “better than” measure, and below the diagonal for the “worse than” measure. The marginal distributions show the Thurstonian model has more mass at very good levels of performance above the 0.9 proportion for the “better than” measure, and more mass below the 0.1 proportion for the “worse than” measure.

## Discussion

The wisdom of the crowd effect is fundamentally about knowledge aggregation. In this paper, we have focused on ranking data as an expression of knowledge, and on cognitive modeling as an approach to aggregation. Ranking data have been considered before in the wisdom of the crowd context [18], but much less often than simple numerical estimates. Cognitive models have been considered before in the wisdom of the crowd context [19], [20], but much less often than standard statistical methods. Very little work has been done that uses a cognitive modeling approach for ranking data [3], and we believe we are the first to use a Thurstonian modeling approach for ranking data that allows for individual differences and involves a known ground truth.

Our results show that it is possible to combine people's rankings in useful ways, across a range of tasks covering general knowledge as well as prediction problems. Both the Thurstonian modeling approach we developed, and the standard Borda count statistical approach, produce combined rankings that perform well relative to individuals. There is some evidence in our results that the Thurstonian modeling approach outperforms the Borda method, although it is far from definitive. Over all of the tasks we considered, the ranking generated by the Thurstonian model usually outperforms the one generated by the Borda method, and more often achieves very good levels of performance relative to individuals. There is also some evidence in our results that the Thurstonian model fares especially well in prediction tasks. In the four prediction tasks we considered, shown in Figure 5, the Thurstonian model outperforms the Borda method, and is among the best of (or better than) the individual participants. Future research needs to consider additional prediction tasks to provide further evaluation of this suggestive finding.

Beyond the assessments provided by performance in matching ground truths, however, we believe our results provide several more general reasons for pursuing the Thurstonian approach. The Thurstonian approach adopts a generative cognitive modeling perspective, in which the focus is on formalizing how latent psychological processes and parameters generate behavioral data. This perspective means the Thurstonian model provides additional information not immediately available from standard statistical methods like the Borda count.

The best example of this general difference is in the measure of individual expertise inferred by our model. As part of modeling how people generate rankings, our model assumes each person has some level of knowledge of the items being ranked. This level of knowledge is parametrized by 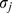 for the 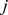
th person, and is naturally interpreted as a measure of their expertise. The scatter-plots in Figures 3, 4, and 5 show that the expectation of the posterior of the expertise parameters correlates, often very highly, with the tau distance measure of actual performance. This is a compelling set of results, because the *the true ordering of the items is not needed nor used* to infer the expertise measures. The strong correlations thus suggests that a person's relative expertise can be measured effectively by the Thurstonian model, using only their behavioral data in completing the ranking task, without any specific form of self-assessment of expertise, and without knowing the ground truth [13].

**Figure 7:**
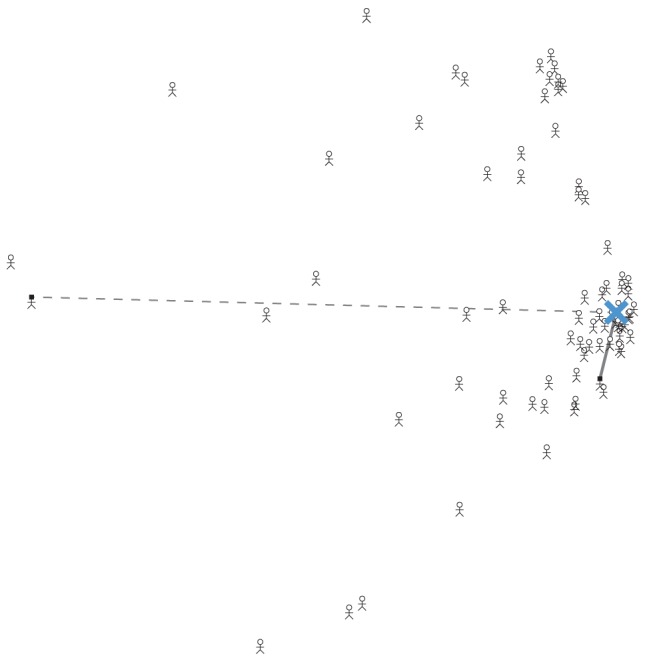
Intuition for successful performance of the Thurstonian approach to modeling the wisdom of crowds. A projected representation of the distances between the rankings provided by participants in the movie rankings task, showing a large cluster of people with similar answers. The ranking found by the Thurstonian model is shown by the large blue cross. An participant inferred to be relatively expert near the Thurstonian ranking is connected by the solid line, and a participant inferred to be relatively non-expert far from the Thurstonian ranking is connected by the broken line.

Figure 7 presents our intuition as to why the Thurstonian model might outperform the Borda method, and is able to infer expertise successfully. The spatial arrangement of participants, shown by stick figures, was found by applying classical multidimensional scaling methods [21] to the tau distances between the rankings for every pair of participants in the movie release dates task. There is a visually clear cluster of participants in the middle-right of this arrangement, all giving similar rankings. The Thurstonian model is able to make this pattern of observed data likely by inferring that the true latent locations of the items are consistent with the rankings in the cluster, and that participants in this cluster have narrow distributions from which they draw their mental samples. Colloquially, the model infers that strong agreement on small set of rankings out of a possible 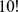 is unlikely to have occurred by chance, but likely to have occurred if the true order is providing a signal shared by these participants.

The result of these inferences is that the aggregate ranking produced by the Thurstonian model will fall in the cluster, as shown by the blue cross, and that the inferred expertise of each participant will depend on how close their ranking is to the aggregate ranking. Figure 7 highlights both a relatively expert participant, connected to the aggregate ranking by the solid line, and a relatively non-expert participants, connected to the aggregate ranking by the broken line. One way of thinking about how the Thurstonian model differs from the Borda count in producing an aggregate ranking is that the Thurstonian model incorporates the inferred expertise. Those participants with narrower distribution (smaller 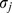 values) will have relatively greater influence on the inference of the latent locations (the 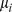 values). In this sense, the Thurstonian model “up-weights” the rankings of relatively expert participants in determining an aggregate ranking, whereas the Borda count method treats the ranking of each participant as having equal weight.

Of course, it would be possible to develop an extended Borda count method that determined an expertise measure by comparing individual and aggregate rankings, and used this measure as a weight in combining individual rankings. Such an approach is likely to perform similarly to the Thurstonian model we have developed, although it would lack the inferential completeness provided by the full joint posterior distribution afforded by the Thurstonian model's Bayesian approach. We note, however, that the intuition that expertise can be measured by proximity to an aggregate answer, and used as a weight in combining rankings, emerged from the generative and cognitive modeling approach used to develop the Thurstonian model. By thinking about how people produce rankings, cognitive processes and parameters were formalized that provided useful new insights, methods, and results.

The cognitive model developed here is a very simple psychological account of how rankings are produced, and it is easy to see multiple complementary ways in which it could be extended. The assumption that there is a single latent true ordering could be relaxed to allow for different substantive opinion, perceptions,or misperceptions. There is a suggestion in Figure 7 of a separate smaller cluster of participants who produced different rankings This sort of representational structure can be modeled as a latent mixture over multiple rankings, and naturally expressed as a generative cognitive model with a richer sort of individual differences. In some applications, one of the latent orders might be regarded as a contaminant (e.g., if there is a common misperception of some aspect of general knowledge), or both latent orders might be regarded as legitimate opinions, as in cultural consensus theory [22]–[24]. One appealing possibility is that the identification of different representations might often be possible, and especially important, in prediction settings. For example, in making the predictions about NBA basketball outcomes, it seems plausible that some significant subset of non-expert participants might base their rankings on criteria like city size or familiarity of team name. Creating a mixture model representation that includes a mixture component capturing these heuristic strategies might allow more precise and useful inferences about the latent representations being used to generate rankings by more expert participants.

A closely related extension is to consider more sophisticated models of individual differences. The current modeling approach assumes that a person's expertise can be represented by a single scalar (the 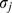 parameter), and that these are independent for each question. Theories of individual differences in expertise [25], and more general psychometric theories—including classic test theory and item-response theory models [26], and factor measurement models [27]—provide a basis for extending this simple assumption. Future work should also examine how structured measures of individual differences and expertise derived from these theories relate to observed co-variates, including measures like age, gender, self-rated expertise, and so on. All of these sorts of extensions are naturally incorporated in the graphical modeling framework, as hierarchical extensions of the current model.

Finally, our Thurstonian model could also be extended to include more realistic memory processes. For example, the identification of Lincoln as the 16th president noted in Figure 2 suggests that people have both relatively and absolute knowledge for order. In terms of the theories of memory needed to model people's behavior, both chaining and position encoding mechanisms are clearly important [28]. Chaining models of memory assume that sequences of items of encoded, and the retrieval of one cues the retrieval of the next (Carter, Reagan, Bush, Clinton, …), while position models assume that item-slot relationships are encoded (Lincoln is the 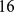
th president). Incorporating these sorts of memory mechanisms into a cognitive model of how people produce ranking data is likely to have implications for how their ranking are aggregated and their individual differences are understood.

The wisdom of the crowd is a fundamental challenge for cognitive modeling. It requires understanding how different people represent the potentially different knowledge they have, and how they express that knowledge in behavioral tasks. We have shown that a simple cognitive model performs well on the problem of combining people's rankings of items, including in predictive settings, and provide useful insights into the expertise of individuals. Future work should develop richer cognitive models, incorporating more sophisticated representation, memory, and decision-making processes, with the complementary aims of improving our applied ability to aggregate ranking data, and our theoretical understanding of the knowledge individuals and groups share.

## Supporting Information

Appendix S1 (PDF)
