# Supplementary material for: A Cognitive Model for Aggregating People's Rankings
Source: PLoS One. 2014 May 9;9(5):e96431. doi: 10.1371/journal.pone.0096431 (PMC4015936; doi:10.1371/journal.pone.0096431)
Supplement: Appendix S1 — (PDF) [file pone.0096431.s001.pdf]

Supporting Information

The behavioral data are the complete rankings provided by m participants for a question involving n items. These data take the form $\boldsymbol{y}_j = (y_{1j}, \dots, y_{nj})$ for the ranking provided by the jth participant, where y_{ij} is the ranking they provide for the ith item. The goal of an aggregation method is to produce a single ranking $\boldsymbol{y}^\star$.

Borda Count Aggregation

In the Borda count method [1], the rankings provided by each participant correspond to points for each item, according to where the item is ranked. Specifically, if the ith item is ranked in the kth position by the jth participant, then the item receives k points. These points are then summed across all of the participants, to give a total $t_i = \sum_{j=1}^{m} y_{ij}$ for the ith item. The aggregate ranking $\boldsymbol{y}^\star$ produced by the Borda count method is simply the ordering of the items according to the t_i totals, with ties broken at random.

Thurstonian Model Aggregation

The Thurstonian model involves parameters $\boldsymbol{\mu} = (\mu_1, \dots, \mu_n)$ for the items and $\boldsymbol{\sigma} = (\sigma_1, \dots, \sigma_m)$ for the participants. As described in the main text, the model assumes the jth person generates a mental sample of the ith item as $x_{ij} \sim \text{Gaussian}\big(\mu_i, \sigma_j\big)$. The observed data then arise from ranking these mental samples, so that the ranking given to the ith item is the rank of x_{ij} among $x_{1j}, \dots, x_{nj}$, so that $y_{ij} = \text{Rank}\big(x_{ij}\big)$

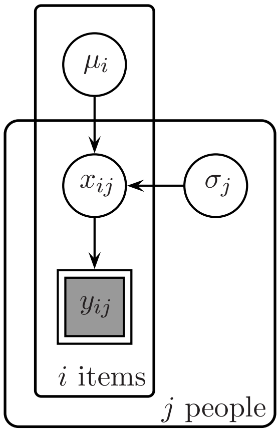

$$
\begin{aligned}
\mu_i &\sim \text{Gaussian}(0, 0.001)\\
\sigma_j &\sim \text{Uniform}(0, 20)\\
x_{ij} &\sim \text{Gaussian}(\mu_i, 1/\sigma_j^2)\\
y_{ij} &\leftarrow \text{Rank}\,(x_{ij})
\end{aligned}
$$

Figure 1. Graphical model representation of the Thurstonian model. The latent item location μ_i for the ith item, and the latent expertise σ_j of the jth person influence the mental sample $x_{ij} \sim \text{Gaussian}\big(\mu_i, 1/\sigma_j^2\big)$. The observed ranking data for the jth person, $\boldsymbol{y}_j$ follow deterministically from the mental samples. Plates show the repetition of the graphical model over the items and people.

A graphical model that formalizes this probabilistic generative process for the data is shown in Figure 1. Graphical models are a standard formalism used in machine learning [2] and cognitive science [3,4]. In graphical models, variables are represented as nodes in a graph, and the graph structure is used to indicate dependencies between variables, with children depending on their parents.

In Figure 1, observed variables—the ranking y_{ij} of the ith item provided by the jth person—are represented by shaded nodes, while unobserved variables—the latent locations μ_i, latent expertise values σ_j and latent mental samples x_{ij}—are represented by unshaded nodes. Continuous variables are shown

by circular nodes, while discrete variables are shown by square nodes. In addition, because the ranking data follow deterministically from the samples, the node for y_{ij} is double-bordered. Finally, enclosing plates for both items and people are used in the graphical model to indicate independent replications of the graph structure.

The following JAGS script implements the graphical model in Figure 1.

```
# Thurstonian Model
data{
  lower <- -10
  upper <-  10
  for (i in 1:n){
    for (j in 1:m){
      ones[i,j] <- 1
    }
  }
}
model{
  # Item location
  for (i in 1:n){
    mu[i] ~ dnorm(0,0.001)
   }
  # Person expertise
  for (j in 1:m){
    sigma[j] ~ dunif(0,20)
    lambda[j] <- pow(sigma[j],-2)
  }
  for (i in 1:n){
    for (j in 1:m){
      # The mental sample for the ith item and jth person
      x[i,j] ~ dnorm(mu[i],lambda[j])
      # If the item is in position 1 it is bigger than lower;
      # If the item is in any other position it is bigger than the
      # sample for the item ranked immediately before it
      bounds[i,j,1] <- equals(y[i,j],1)*lower
                       +  inprod(x[i,1:n],equals(y[i,1:n],y[i,j]-1))
      # If the item is in position n it is smaller than upper;
      # If the item is in any other position it is smaller than the
      # sample for the item ranked immediately after it
      bounds[i,j,2] <- equals(y[i,j],n)*upper
                       + inprod(x[i,1:n],equals(y[i,1:n],y[i,j]+1))
      # Censored sampling
      ones[i,j] ~ dinterval(x[i,j],bounds[i,j,1:2])
    }
  }
  # Aggregate ranking
  ystar <- rank(mu[1:n])
}
```

Each MCMC sample contains jointly inferred values for $\mu_1, \ldots, \mu_n$, and the order of these values corresponds to a sample from the posterior of the aggregate order $\boldsymbol{y}^{\star}$. The distributions of tau distances

for the Thurstonian model shown in Figures 3, 4, and 5 correspond to the tau distances from each posterior sample from $\boldsymbol{y}^\star$. The posterior distributions for $\boldsymbol{\mu}$ shown in Figure 2 require, in practice, post-processing of the posterior samples to achieve location and scale invariance. In theory, because the priors on μ_i and σ_j are not entirely uninformative, the statistical problem is well posed without the need for post-processing. In practice, the priors are so weakly informative that post-processing is needed for inferences based on their posterior distributions. The $\hat{R}$ convergence statistic is taken with respect to the posterior distribution of $\boldsymbol{y}^\star$ over chains, since the rankings of the μ_i values are not affected by location and scale.

References

1. Marden JI (1995) Analyzing and Modeling Rank Data. Chapman & Hall.

2. Koller D, Friedman N, Getoor L, Taskar B (2007) Graphical models in a nutshell. In: Getoor L, Taskar B, editors, Introduction to Statistical Relational Learning, Cambridge, MA: MIT Press.

3. Lee MD, Wagenmakers EJ (2013) Bayesian Cognitive Modeling: A Practical Course. Cambridge University Press.

4. Shiffrin RM, Lee MD, Kim WJ, Wagenmakers EJ (2008) A survey of model evaluation approaches with a tutorial on hierarchical Bayesian methods. Cognitive Science 32: 1248–1284.
